# Institutional Amenities

**Published:** 1898-11-12

**Authors:** 


### The Institutional Workshop.

### INSTITUTIONAL AMENITIES.

By a Member of the London Press.

### III.—THEIR "BUSINESS END."

Now that I have stated the facts and theories on which my contention rests, I should like to consider briefly the cure for the state of things which undoubtedly exists in some of our hospitals. I am fully aware that hospital officials are very hard worked—I have been an institutional official myself, and so I can the more readily sympathise with that state of weariness which suggests that one more visitor will be the last straw to break down our composure; and I am equally conscious that many visitors have little consideration for tired officials, and have no conception that "business hours" should prevail in hospitals as elsewhere, and that off-times should not be broken into unless in case of urgency. Holding such views, I have invariably made it a rule when calling at hospitals to do so between the hours of ten and four, which are reasonable hours for doing business; and I have at all times specially avoided a call during the lunch hour, for officials—despite a prevailing view that philanthropic workers "don't mind any sacrifice for their work"—need lunch like other people. I am alive, too, to the necessity for the matron to do a daily round of the wards, and during this time she cannot and should not be—unless the hospital is very small—at the disposal of stray callers. The secretary, too, is a busy person, who needs occasional "close times" for preparing balance-sheets, appeals to the public, reports to committees, &c. Consequently the permanent officials cannot invariably be ready with leisure and courtesy at the command of the chance visitor. But would it not be possible to arrange some system whereby some one person in authority were available to visitors and inquirers from ten to four daily? The secretary might undertake to interview callers one day in the week. The matron might include this among her duties on another day. The assistant matron and assistant secretary, if such exist, might each have the duty deputed to them on a specified day. This would leave only two days in the week to be filled up, and on such days surely some ladies or gentlemen interested in the hospital could be found to take up so pleasant and easy a task as the interviewing of those who might ultimately prove helpful and powerful friends of an institution. In several United States hospitals I have found some such system at work. A Ladies' Committee, consisting of perhaps some 20 individuals, has undertaken that one of their number should be "on duty" daily at the hospital, to fulfil, as it were, that social function so essential to the success of all charitable institutions—as of other businesses. In all business offices there is somebody "on duty" during City hours to interview the caller, to answer inquiries, and give business information; and, as a matter of routine, there is invariably somebody to make himself agreeable to friends of the firm.

There is scope in our hospitals and institutions for the position of interviewer and entertainer of visitors and callers. I should like to bear testimony to the universal and unvarying courtesy of officials at our insane asylums. I have never experienced or heard of an instance where callers at asylums received anything but the most polite and kindly consideration; and the same is true of our prisons, from the governor down to the doorkeeper. But the same happy state of things does not obtain in all our hospitals, and, to my mind, the fact is deplorable. As a question of interest, apart from an ethical courtesy which has no "business end," it pays an institution dependent on contributions to gather interested friends around its portals. Friends mean subscriptions, legacies, and donations in kind as well as in hard cash. Of my own personal knowledge I could cite many instances of generous strangers and persons of influence who have been alienated from an institution by being met at the door with a want of kindliness and consideration. People are very human after all, and they give from a kindly impressionable influence as much as from a stern sense of civic duty. And it is a pleasant circumstance when visiting an institution to be welcomed in a hospitable and cheering spirit, to be ushered into a warm reception-room where there is a well-mannered official, willing to give information in a courteous, agreeable spirit. The secretary of a somewhat small hospital told me recently that its matron—whose manners are exceptionally charming, and who makes a point of being a good hostess to passing visitors—never fails to collect £500 a year for the hospital funds. And this end is accomplished by pleasing and interesting visitors, and not by an uncomfortable system of begging. The chance caller spends a pleasant time at the hospital, and sees all that is interesting. The natural outcome is that generous British impulse to make a return. And the most obvious return is a subscription to the hospital. I feel assured that this question of institutional amenities has its commercial side, for I have noted time and again, both in my British and foreign peregrinations, that where the officials make a point of treating everybody who comes to their institution with courtesy and kindness, a certain prosperity and well-being is the very natural result. Were I secretary or matron of a hospital—poor or otherwise—I should encourage visitors of all sorts and conditions. I would strive to interest everybody of every class in my institution. Pleasant-mannered nurses should show visitors around the wards. There should be a comfortable reception room for callers, and at all times it should be "somebody's business" to make each visitor feel welcome. The good offices of a ladies' committee should be employed, and one of their number should be asked to come to the hospital each day to interest herself in making the hospital interesting to the public. There are many lady visitors connected with hospitals who would do such work admirably. The public supports the hospitals, has a vested interest in their success, and is clearly entitled to a pleasant reception, and an investigation of the good works that they are paying to have done. And the hospital which best recognises such an obvious right and wish on the part of the public, and that sets out to make the inspection an enjoyable occasion, is the hospital which is going to succeed, and which deserves to succeed. I feel less diffident on this part of my subject since I have myself worked very hard in the interests of more than one hospital badly in need of funds, and I found then—as doubtless I should find now, were I again in such a position—that, though the public appeal has its value, the social and individual side is infinitely more efficacious in securing donations and legacies. A pleasant walk round the hospital wards, with a few words in season as to obvious and hidden needs, little shabbinesses, wards without pianos, cots empty from want of funds, &c.; all these pathetic object-lessons which a person sees with his own eyes are more persuasive than a statement in cold print. Indeed, I am firmly convinced that a lack of institutional amenities, and a want of a more social side to our hospitals, plays an important part in a dearth of institutional subscriptions. It is a bad sign when one hears people saying, as they do about sundry hospitals one could name, "I should never think of going there again, I felt such an intruder. Nobody had time to speak to me or to show me the wards. My visit was very uninteresting," &c. The person who is made to feel like an intruder at a hospital is never likely to "intrude" a subscription. And this, after all, is the practical business end of institutional amenities.

